# A few basic concepts in electrochemical carbon dioxide reduction

**DOI:** 10.1038/s41467-020-19369-6

**Published:** 2020-11-23

**Authors:** Karen Chan

**Affiliations:** Department of Physics, Technical University of Denmark, 2800 Kongens Lyngby, Denmark

**Keywords:** Electrocatalysis, Energy

## Abstract

In this perspective, I discuss a few basic concepts in fundamental mechanistic studies of electrochemical carbon dioxide reduction.

## Abstract (web summary)

Electrochemical carbon dioxide (CO_2_) reduction has the potential to sustainably produce carbon-based fuels and chemicals while mitigating the increasing levels of CO_2_ in the atmosphere. In this comment, the author discusses a few basic concepts in the fundamental mechanistic studies of electrochemical CO_2_ reduction.

With the looming global environmental crisis, electrochemical CO_2_ reduction (CO_2_R) is a hot topic. Excellent perspectives on mechanistic studies^1–3^, practical vapor-fed devices^4^, and technoeconomic and system-level analyses^5–7^ have come out in the past few years, all with compelling visions for the future. We can also harken back to Hori’s timeless review of his work over several decades^8^, which seeded many of the impressive advances today. But as the French maxim says: *parfois, il faut reculer pour mieux sauter*. Here, I showcase a few basic concepts in the fundamental mechanistic studies of CO_2_R.

## What computational electrocatalysis can and cannot do

In heterogeneous catalysis, periodic density functional theory (DFT) simulations have really enabled us to computationally explore reaction mechanisms. For electrocatalysis, the “computational hydrogen electrode” model is our standard method to determine reaction thermodynamics^9^. This method trivially translates simulations in vacuum to potential-dependent energetics, without requiring we simulate explicitly the ions or potential.

**Fig. 1:**
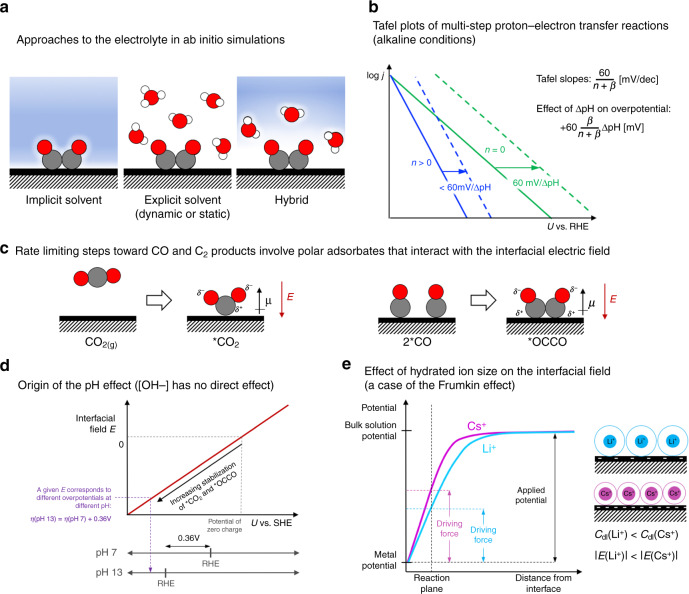
Some basic ideas from mechanistic studies of electrochemical carbon dioxide reduction. **a** Implicit, explicit, and hybrid approaches to model the electrolyte in ab initio simulations. **b** In electrochemical reactions with multiple proton–electron transfers, the number of electrons *n* transferred prior to the rate-limiting step determines both the Tafel slope and how changes in pH shift the activity. **c** Rate-limiting steps for CO_2_ reduction to CO (on weak binding catalysts) and C_2_ products (on Cu) involve intermediates with large dipole moments *μ*, which interact with the interfacial electric field *E*. **d** The absolute potential (e.g., *U* vs. SHE) determines the electric field at an electrochemical interface and the corresponding stabilization of the polar *CO_2_ and *OCCO intermediates. Since the dipoles point away from the interface, the field-stabilization occurs at potentials below the potential of zero charge. A given field stabilization corresponds to a more *positive* overpotential at higher pH (e.g., a 360 mV shift between pH 7 and 13), which leads to higher CO_(2)_R activity at higher pH. **e** The differences in hydrated cation sizes (e.g. hydrated Li^+^ vs. hydrated Cs^+^) lead to differences in the surface charge density and interfacial field at a given applied potential. This model is an example of the Frumkin effect: the interfacial field (or equivalently the local potential drop) is the driving force for electrochemical processes, and different compositions of the electric double layer give rise to different fields at a given applied potential.

Our models of the electrolyte and electrochemical reaction barriers, in contrast, are far from convergent. Our field abounds with different approaches towards the electrolyte: implicit continuum models, explicit ab initio ones, or a hybrid of the two (Fig. 1a)^10^. We also have multiple ways to obtain the potential and the potential dependence of the reaction energetics^11^. While continuum approximations give us huge reductions in computational cost, we see significant deviations in solvation energies determined with implicit vs. dynamic explicit water models^12^. Furthermore, different ways to set up the applied potential result in differences in the computed reaction energetics^13^. All these challenges could contribute to the wide range in the computed energetics and mechanisms towards the various C_2_ products^1^.

Despite the difficulties in an ab initio treatment of electrochemical reaction barriers, we do need kinetics for mechanistic understanding. Case in point: our evolving understanding of CO_(2)_R to CH_4_ on Cu^14^. A thermodynamic analysis showed a proton–electron transfer to *CO to form *CHO to be the rate-limiting step. Considering the corresponding barriers across different materials, we suggested the transition state of this process to be the descriptor of activity. But when we simply consider the kinetics of electrochemical reactions with multiple proton–electron transfers, we see that *this step cannot be rate limiting on Cu*. The corresponding Tafel plots have defining features that depend on the symmetry factor *β* (0 < *β* < 1) for the rate-limiting step, as well as the number of proton–electron transfers preceding it, *n.* Figure 1b shows the Tafel slopes and the effect of pH on the overpotential for alkaline solutions, where H_2_O as the proton donor. Experiments show CH_4_ activity to have a Tafel slope of <60 mV/dec, and the positive shift in overpotential with an increased pH is <60 mV/pH unit, both consistent with *n* > 0. Therefore, the rate-limiting step must occur *after the initial proton–electron transfer* to *CO to form *CHO (or *COH^15^) . And Hori, in fact, made these observations decades ago^8^.

To put some numbers on what we can do today, consider the Arrhenius law,

1 $${\mathrm{TOF}} = A\exp \left( { - \frac{{E_{\mathrm{a}}}}{{kT}}} \right),$$

where *A* is the prefactor, TOF the turnover frequency, and *E*_a_ the activation energy. Without even considering the electrochemical environment, a typical DFT error in adsorption energy is 0.15 eV ^16^. A shift in *E*_a_ of this magnitude gives a 300× change in the TOF at room temperature! Depending on the reaction process at play, the corresponding theoretical selectivities can have uncertainties that approach 100%^17^. With electrochemical barriers, the uncertainties are compounded by the challenges mentioned above.

So DFT-based kinetic models do not presently give us predictions of activity or selectivity to the precision of experiments where mass transport and the surface structure of the catalyst are carefully controlled. With error cancellation, we have much more confidence in the *relative*: the relative magnitudes of barriers within a given mechanism, as well as the relative activity across catalysts^16^ and across reaction conditions^18^. Especially with our present degree of accuracy, we should, wherever possible, couple DFT models to ample feedback from experiment. Such joint efforts have given us valuable insights into reaction mechanisms, activity descriptors, and electrolyte effects^1^.

## The activity towards CO and C_2_ products is driven by field–dipole interactions

A special feature of CO_(2)_R is the importance of steps that *do not involve a proton–electron transfer* (Fig. 1c). On weak binding catalysts (e.g. Au^19^ and Fe-N-C^20^ catalysts), the rate of CO_2_R to CO is limited by CO_2_ adsorption. On Cu catalysts, CO–CO coupling limits the rate of CO_(2)_ reduction to high-value C_2_ products such as ethanol and ethylene^3^.

Now, the dipoles of *CO_2_ and *OCCO interact strongly with the interfacial electric field. And since the field depends on the *absolute* potential, e.g. on an SHE scale, so does the activity of the corresponding reactions. Figure 1d shows the variation of the interfacial field *E* and the corresponding stabilization of the polar intermediates vs. the potential *U* vs. SHE. On the other hand, the overpotential *η* depends on *U* vs. RHE, which shifts on the SHE scale by a Nernstian factor of 60 mV/pH unit. For reduction reactions, a positive shift in *η* translates to higher activity. For example, a shift in pH from 7 to 13 translates to a whopping shift in *η* of +0.36 V! We can therefore think of the dramatic pH effect for these products as simply arising from a shift in the RHE reference potential.

We can use different labels for this phenomenon, such as single electron transfer^19^ or decoupled proton–electron transfer^3^, but the dipole-field vocabulary allows us to consider the reaction rate in terms of dipoles of the intermediates, *μ*, and the interfacial capacitance, *C*_dl_. For example, the rate of CO_2_ adsorption and the corresponding Tafel slope are as follows^21,22^:

2 $$j \propto \exp \left( { - \frac{{\mu \left( {{\mathrm{CO}}_2} \right)_{{\mathrm{TS}}} \cdot E}}{{kT}}} \right),$$

3 $${\mathrm{Tafel}}\,{\mathrm{slope}} = \left| {\frac{{\partial U}}{{\partial \log j}}} \right| \propto \frac{1}{{\mu \left( {{\mathrm{CO}}_2} \right)_{{\mathrm{TS}}}C_{{\mathrm{dl}}}}}$$

and we can write analogous expressions for CO dimerization.

Note that the local [OH^–^], which increases with increasing CO_(2)_R current, *plays no direct role in promoting the rate of these two steps*^23^*, since they are driven by the field alone*. However, the [OH^–^] *can* alter the CO_2_ concentration through the bicarbonate equilibria, suppress CH_4_ formation^14^, and promote the activity towards acetate, even at a fixed *U* vs. SHE^24^.

These very dipole–field interactions also rationalize the sensitivity of activity to cation identity^18^ (Fig. 1e). In a classical picture of the interface, the ion concentration is limited by the hydrated ion size^25^. The smaller the size, the greater the surface charge and interfacial field for a given applied potential, which increases *C*_dl_. The slightly *smaller* hydrated size of Cs^+^ vs. Li^+^ leads to the 1–2 orders of magnitude enhancement for the CO activity on Ag and C_2_ activity on Cu.

This model of the ion effects is an echo of the decades-old “Frumkin diffuse layer correction” to Butler–Volmer kinetics^26^. This correction accounts for the impact of the composition of the double layer on the *local* potential drop, which determines the corresponding reaction rate. Beyond electrostatics, specific chemical interactions between ions with the surface or adsorbate may also play a role, and both cations and anions can act as buffers^27,28^.

The dependence of CO_(2)_R on adsorbate–field interactions shows us that, in addition to optimizing the adsorption energies of critical intermediates, we can look to tuning *C*_dl_ and *μ* towards higher activity (Eq. 3). Our models suggest that we can tune the former through the electrolyte, and the latter in single-atom catalysts, where the localization of charge on the active site is affected by the coordinating atoms^22^.

## We need TOF estimates to evaluate intrinsic activity, and Cu’s still the best (but don’t give up)

What do we know about the activity of existing catalysts? Selectivities are often represented by Faradaic efficiencies: ${\mathrm{FE}}_{\mathrm{i}} = \frac{{j_i}}{{j_{{\mathrm{{tot}}}}}}$, where *j*_*i*_ is the partial current density of product *i* and *j*_tot_ the total current density. While selectivities are a critical performance metric, FE_*i*_*’s can’t be used to evaluate the intrinsic activity* towards a given product, especially as they shift with respect to changes in the activities of all other products. *The intrinsic activity, as determined by the reaction energetics, can really only be evaluated by TOFs* (Eq. 1). In practice, we approximate TOFs by partial current densities normalized to the electrochemically active surface area (ECSA), $j_{{\mathrm{{ECSA}}}} \propto \rho _{{\mathrm{{site}}}}{\mathrm{TOF}}$, where *ρ*_site_ is the density of the active site. Comparisons of intrinsic activity with *j*_ECSA_ are therefore accurate within the variations of *ρ*_site_ among samples, the uncertainty in the ECSA, and the degree of mass transport limitations.

Surface reaction energetics on different facets typically differ by 0.1–1 eV ^29^, which translates to variations in the corresponding TOFs *by orders of magnitude* (Eq. 1). Shifts in *j*_ECSA_ of around an order of magnitude (or less) between catalysts with different surface structures are more likely to arise from a change in *ρ*_site_ than a change in the predominant active site or facet. Recent reviews have shown that nano-structured Cu and Cu-based bimetallics show similar *j*_ECSA_ to those on Cu foils^1,2^. To date, I am not aware of a new catalyst with *intrinsic activity* towards C_2_ products that unequivocally exceeds that of Cu foil. Ongoing efforts to obtain single crystal measurements with product quantification can rigorously evaluate theoretical predictions of the most active Cu facet(s).

The increased C_2_ selectivities on various high surface area Cu catalysts actually arise from the *suppression* of other products, such as CH_4_ and H_2_^1,2^. Under alkaline conditions, H_2_ suppression cannot arise from local changes in pH, since H_2_O is the proton donor. Perhaps nanostructuring shifts the structure and activity of water, such that products that are limited by proton–electron transfer steps are suppressed.

And why haven’t we found alternatives to Cu that either match or exceed its intrinsic activity towards C_2_ products? Stability is a possible culprit: leaching or surface restructuring, which can be driven by the presence of elements that strongly bind *CO. But I know no fundamental limitation on the existence of stable and active alternatives, especially if we expand our search to emergent classes of materials beyond binary combinations of transition metals^30,31^. Furthermore, improvements in catalytic efficiency are still needed^5,6^. With a rigorous consideration of surface stability, the discovery of new catalysts beyond Cu remains a worthwhile and important pursuit.

## Outlook

Even as we develop practical devices and systems for CO_2_R, we still face fundamental challenges at the level of reaction mechanisms and intrinsic activity. These challenges range from simulating electrochemical kinetics to the discovery of new catalysts beyond Cu. Tremendous opportunity lies in overcoming them. With the increasing dialogue among us and the diversity of expertise we are bringing together—I envision that our collective efforts will ultimately contribute to establishing a sustainable carbon cycle.

## References

[1] Nitopi S (2019). Progress and perspectives of electrochemical CO2 reduction on copper in aqueous electrolyte. Chem. Rev..

[2] Resasco J, Bell AT (2020). Electrocatalytic CO2 reduction to fuels: progress and opportunities.. Trends Chem..

[3] Kortlever R, Shen J, Schouten KJP, Calle-Vallejo F, Koper MTMM (2015). Catalysts and reaction pathways for the electrochemical reduction of carbon dioxide. J. Phys. Chem. Lett..

[4] Higgins D, Hahn C, Xiang C, Jaramillo TF, Weber AZ (2019). Gas-diffusion electrodes for carbon dioxide reduction: a new paradigm. ACS Energy Lett..

[5] Jouny M, Luc W, Jiao F (2018). General techno-economic analysis of CO_2_ electrolysis systems. Ind. Eng. Chem. Res..

[6] Verma S (2016). A gross-margin model for defining technoeconomic benchmarks in the electroreduction of CO_2_. ChemSusChem.

[7] Smith WA, Burdyny T, Vermaas DA, Geerlings H (2019). Pathways to industrial-scale fuel out of thin air from CO_2_ electrolysis. Joule.

[8] Hori, Y. In *Modern Aspects of Electrochemistry* (eds Vayenas, C. G. et al.) 89–189 (Springer, New York, 2008).

[9] Nørskov JK (2004). Origin of the overpotential for oxygen reduction at a fuel-cell cathode. J. Phys. Chem. B.

[10] Schwarz K, Sundararaman R (2020). The electrochemical interface in first-principles calculations. Surf. Sci. Rep..

[11] Groß A (2020). Theory of solid/electrolyte interfaces. Surf. Interface Sci..

[12] Heenen HH, Gauthier JA, Kristoffersen HH, Ludwig T, Chan K (2020). Solvation at metal/water interfaces: an ab initio molecular dynamics benchmark of common computational approaches. J. Chem. Phys..

[13] Gauthier JA (2019). Unified approach to implicit and explicit solvent simulations of electrochemical reaction energetics. J. Chem. Theory Comput..

[14] Liu X (2019). pH effects on the electrochemical reduction of CO2 towards C2+ products on stepped copper. Nat. Commun..

[15] Nie X, Esopi MR, Janik MJ, Asthagiri A (2013). Selectivity of CO_2_ reduction on copper electrodes: the role of the kinetics of elementary steps. Angew. Chem. Int. Ed..

[16] Medford AJ (2014). Assessing the reliability of calculated catalytic ammonia synthesis rates. Science.

[17] Yang N (2016). Intrinsic selectivity and structure sensitivity of rhodium catalysts for C_2+_ oxygenate production. J. Am. Chem. Soc..

[18] Ringe S (2019). Understanding cation effects in electrochemical CO2 reduction. Energy Environ. Sci..

[19] Wuttig A, Yaguchi M, Motobayashi K, Osawa M, Surendranath Y (2016). Inhibited proton transfer enhances Au-catalyzed CO_2_-to-fuels selectivity. Proc. Natl Acad. Sci. USA.

[20] Varela AS (2018). pH Effects on the selectivity of the electrocatalytic CO_2_ reduction on graphene-embedded Fe–N–C motifs: bridging concepts between molecular homogeneous and solid-state heterogeneous catalysts. ACS Energy Lett..

[21] Ringe S (2020). Double layer charging driven carbon dioxide adsorption limits the rate of electrochemical carbon dioxide reduction on gold. Nat. Commun..

[22] Vijay S (2020). Dipole-field interactions determine the CO_2_ reduction activity of 2D Fe-N-C single atom catalysts. ACS Catal..

[23] Li J (2020). Hydroxide is not a promoter of C_2+_ product formation in the electrochemical reduction of CO on copper. Angew. Chem. Int. Ed..

[24] Luc W (2019). Two-dimensional copper nanosheets for electrochemical reduction of carbon monoxide to acetate. Nat. Catal..

[25] Borukhov I, Andelman D, Orland H (2000). Adsorption of large ions from an electrolyte solution: a modified Poisson–Boltzmann equation. Electrochim. Acta.

[26] Bard, A. J. & Faulkner, L. R. *Electrochemical Methods: Fundamentals and Applications,* 2nd edn (John Wiley & Sons, 2000).

[27] Singh MR, Kwon Y, Lum Y, Ager JW, Bell AT (2016). Hydrolysis of electrolyte cations enhances the electrochemical reduction of CO_2_ over Ag and Cu. J. Am. Chem. Soc..

[28] Zhang F, Co AC (2020). Direct evidence of local pH change and the role of alkali cation during CO2 electroreduction in aqueous media. Angew. Chemie Int. Ed..

[29] Nørskov, J. K., Studt, F., Abild-Pedersen, F. & Bligaard, T. *Fundamental Concepts in Heterogeneous Catalysis* (John Wiley and Sons, 2014).

[30] Piontek S (2019). Bio-inspired design: bulk iron-nickel sulfide allows for efficient solvent-dependent CO_2_ reduction. Chem. Sci..

[31] Pedersen JK, Batchelor TAA, Bagger A, Rossmeisl J (2020). High-entropy alloys as catalysts for the CO_2_ and CO reduction reactions. ACS Catal..

